# Inhibition of breast cancer resistance protein at the murine blood-brain barrier by Ko143 studied with [^11^C]tariquidar and PET

**DOI:** 10.1186/1471-2210-11-S2-A48

**Published:** 2011-09-05

**Authors:** Thomas Wanek, Claudia Kuntner, Jens P Bankstahl, Marion Bankstahl, Johann Stanek, Michael Sauberer, Markus Müller, Wolfgang Löscher, Oliver Langer

**Affiliations:** 1 Health and Environment Department, Molecular Medicine, AIT Austrian Institute of Technology GmbH, 2444 Seibersdorf, Austria; 2 Department of Pharmacology, Toxicology and Pharmacy, University of Veterinary Medicine, and Center for Systems Neuroscience, 30559 Hannover, Germany; 3 Department of Clinical Pharmacology, Medical University of Vienna, 1090 Vienna, Austria

## Background

The ATP-binding cassette (ABC) transporters breast cancer resistance protein (BCRP) and P-glycoprotein (P-gp) are expressed in the blood-brain barrier (BBB), where they impede brain uptake of their substrates by active efflux transport. BCRP has recently been shown to be the quantitatively most important ABC transporter at the human BBB. Inhibition of BCRP by inhibitors such as the fumitremorgin C derivative Ko143 [1] may be an interesting strategy to improve brain uptake of BCRP substrates. The aim of this study was to assess the dose-response relationship of Ko143 for inhibition of Bcrp1 at the murine BBB using small-animal positron emission tomography (PET) together with the dual P-gp/BCRP substrate radiotracer [^11^C]tariquidar.

## Methods

[^11^C]Tariquidar PET scans were performed in female wild-type (FVB), *Bcrp1*^−/−^ and *Mdr1a/b*^−/−^ mice before and 60 min after i.v. injection of Ko143 (Axon Medchem BV, The Netherlands) at a dose of 5 mg/kg. Additionally, in *Mdr1a/b*^−/−^ mice scans were performed after i.v. administration of vehicle (n = 2), 1 mg/kg (n = 2), 3 mg/kg (n = 1), 10 mg/kg (n = 3) and 15 mg/kg (n = 2) of Ko143. After the 60-min PET scans a venous blood sample was taken by retro-orbital puncture. Brains were manually outlined on the reconstructed PET images and time-activity curves expressed as percent injected dose per gram (%ID/g) were generated, for which areas under the curve (AUC) were calculated.

## Results

Wild-type and *Bcrp1*^−/−^ mice showed no increase in brain AUCs after administration of 5 mg/kg Ko143 as compared to baseline scans, whereas in *Mdr1a/b*^−/−^ mice brain AUC was 4.5-fold increased. In *Mdr1a/b*^−/−^ mice, the half-maximum effect dose of Ko143 to increase brain AUC of [^11^C]tariquidar was 5.6 ± 2.3 mg/kg. Maximum increase in brain AUC was 8.2-fold after the 15 mg/kg dose. No changes in blood activity concentrations of [^11^C]tariquidar were found after administration of different Ko143 doses.

## Conclusions

Performing PET scans in *Mdr1a/b*^(-/-)^ mice in combination with the dual P-gp/BCRP substrate [^11^C]tariquidar allowed individual assessment of Bcrp1 inhibition at the BBB. Our data demonstrate that Ko143 is a potent inhibitor of cerebral Bcrp1 *in vivo*, which apparently does not inhibit P-gp at the studied doses.
